# Hydrometallurgical Recovery of Metals from Large Printed Circuit Board Pieces

**DOI:** 10.1038/srep14574

**Published:** 2015-09-29

**Authors:** U. Jadhav, H. Hocheng

**Affiliations:** 1 Department of Power Mechanical Engineering, National Tsing Hua University, No. 101, Sec. 2, Kuang Fu Rd., 30013, Hsinchu, Taiwan ROC

## Abstract

The recovery of precious metals from waste printed circuit boards (PCBs) is an effective recycling process. This paper presents a promising hydrometallurgical process to recover precious metals from waste PCBs. To simplify the metal leaching process, large pieces of PCBs were used instead of a pulverized sample. The chemical coating present on the PCBs was removed by sodium hydroxide (NaOH) treatment prior to the hydrometallurgical treatment. Among the leaching reagents examined, hydrochloric acid (HCl) showed great potential for the recovery of metals. The HCl-mediated leaching of waste PCBs was investigated over a range of conditions. Increasing the acid concentration decreased the time required for complete metal recovery. The shaking speed showed a pronounced positive effect on metal recovery, but the temperature showed an insignificant effect. The results showed that 1 M HCl recovered all of the metals from 4 cm × 4 cm PCBs at room temperature and 150 rpm shaking speed in 22 h.

The production of electric and electronic equipment (EEE) is rapidly increasing due to the revolution of informatics technology^1^. A variety of equipment is classified as EEE^2^. Printed circuit boards (PCBs) are essential components of almost all waste EEE^3^. Technological advancements in EEE have shortened their life span, thus causing a huge tonnage of waste PCBs to be produced, which presents a new environmental challenge^3^,^4^,^5^. The metal content of waste PCBs can be as high as 40%^6^, and such metals typically include Cu, Sn, Pb, Cd, Cr, Zn, Ni, and Mn^1^. Therefore, one special target for waste EEE recycling is PCBs. These waste PCBs can be a rich secondary source of valuable metals. Recycling waste PCBs is useful for not only resource recovery but also protecting the environment^7^.

Several methods based on pyrometallurgy^8^,^9^ and hydrometallurgy^1^,^10^ are currently used for the recovery of metals from waste PCBs. Pyrometallurgical processes require heating the waste EEE at high temperatures to recover valuable metals. These treatments lead to the production of hazardous gases that must be removed from the air with flue gas cleaning systems. These processes are energy intensive and high-cost and require high-grade (rich in copper and precious metals) feeds. The formation of dioxins and furans is unavoidable due to the use of halogenated flame retardants, which presents environmental problems; thus, off-gas treatment is a prerequisite^1^,^11^. Compared with pyrometallurgical processes, hydrometallurgical processes offer a relatively low capital cost, reduced environmental impact and high metal recoveries. These processes are relatively suitable for small-scale applications. These attributes make the hydrometallurgical process a potential alternative for the treatment of waste EEE^1^,^7^,^12^. Hydrometallurgical processes involve the dissolution of metals in alkaline or acid medium. Several studies have reported the use of nitric acid (HNO_3_), HCl, sulfuric acid (H_2_SO_4_) and aqua regia for the recovery of metals from waste PCBs^1^,^13^,^14^,^15^. Reagents such as cyanide^16^, halide^17^, thiosulfate^18^, and thiourea^19^ have also been commonly used for the recovery of precious metals. Most of the above-mentioned reports used powdered/pulverized waste PCBs for metal recovery. However, little is known about the use of large pieces of PCBs for the recovery of metals. The effect of the above-mentioned leaching reagents on the PCB pieces during the hydrometallurgical recycling process has also not been reported. If complete metal recovery can be achieved from large pieces of PCBs, then the remaining board (nonmetallic part) can be easily recycled, which would be difficult if pulverized PCBs were used. In this study, we propose an efficient hydrometallurgical process using large pieces of PCBs rather than pulverized PCBs to simplify the overall recycling (metallic as well as nonmetallic fraction) process. In the proposed process, the waste PCBs were pre-treated to remove the chemical coating present. Then, the PCBs were subjected to an acid leaching process for metal recovery. In the present study, various process parameters were optimized for metal recovery.

## Results and Discussion

**Figure 1:**
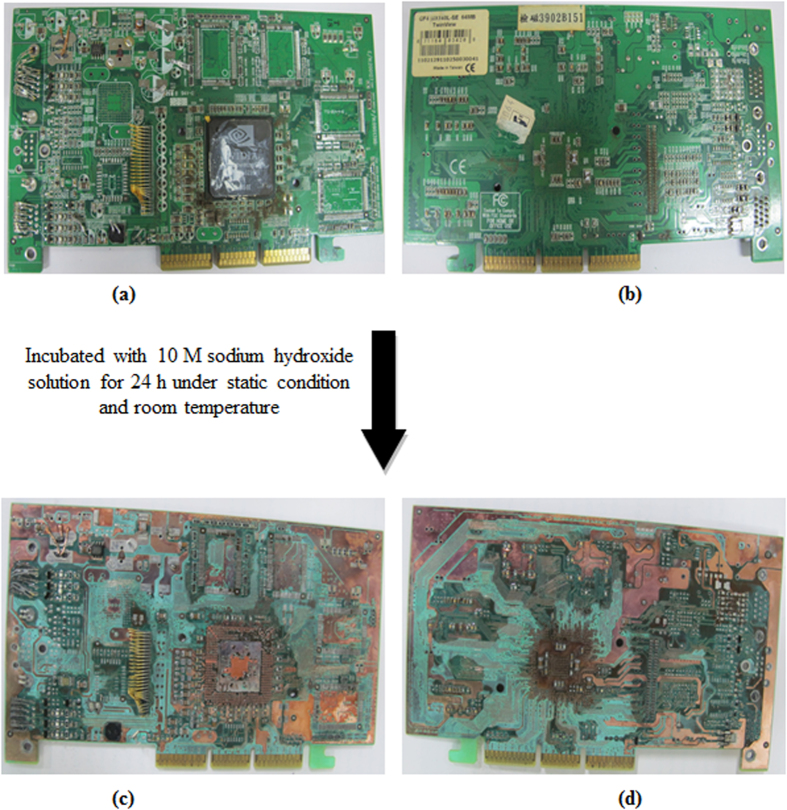
Removal of chemical coating from PCB, before sodium hydroxide treatment [(a) front side of PCB, (b) back side of PCB] and after sodium hydroxide treatment [(c) front side of PCB, (d) back side of PCB].

The metals present on the PCB are covered by a chemical coating^3^,^20^,^21^. This makes metal recovery complicated because the chemical coating inhibits the contact between a lixiviant and the metal. Therefore, it is necessary to first remove as much of the chemical coating as possible. At present, a mechanical–physical method is widely employed to treat waste PCBs and separate the metal and nonmetal^3^,^20^. One important liability of physical separation processes is a significant loss of valuable metals. The reasons for these losses include the insufficient liberation of metals due to their intimate association with plastics, the generation of fines during size reduction and the inefficiency of separation processes for metal recovery from fine fractions^1^. Therefore, it is necessary to find an alternative process for removing the chemical coatings from PCBs without loss of metals. Adhapure *et al.*^21^ used sodium hydroxide to remove the chemical coating. A similar process was used in the present study to remove the chemical coating. Figure 1 shows a PCB piece before and after NaOH treatment. The chemical coating on the PCB piece was completely removed. The samples were analyzed for metal content after the NaOH treatment. ICP analysis show that during the NaOH treatment, significant amount of Al dissolved. Around 911 (±0.85) μg/g Al was leached. At the same time, around 27 (±0.10), 21 (±0.04), 10 (±0.370), 4 (±0.02), 0.43 (±0.001) and 0.11 (±0.003) μg/g Zn, Sn, Fe, Pb, Cu and Ni were leached, respectively. No dissolution of Ag, Pd and Au metals was observed (data not shown). The amounts of Zn and Pb leached were very small compared with the average metal content of the PCB (Table 1). After NaOH treatment, the PCBs were processed further for the hydrometallurgical recovery of metals.

**Figure 2:**
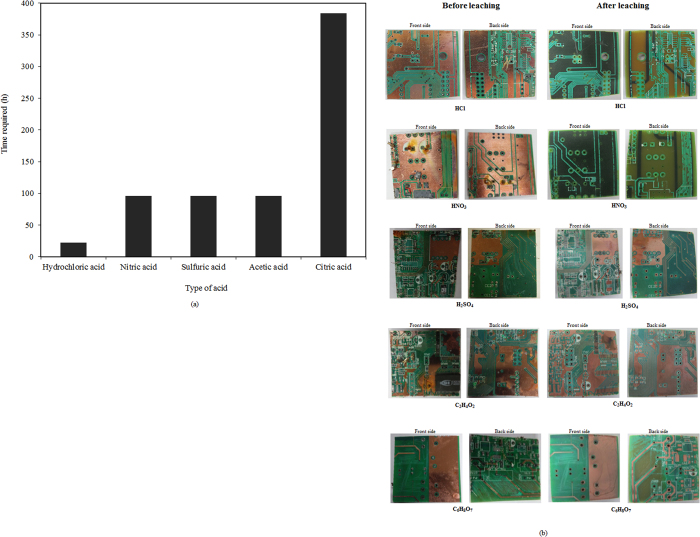
Effect of various lixiviants on (a) the time required for metal recovery from the PCB pieces and (b) the surface appearance of the PCB pieces before and after leaching.

**Figure 3:**
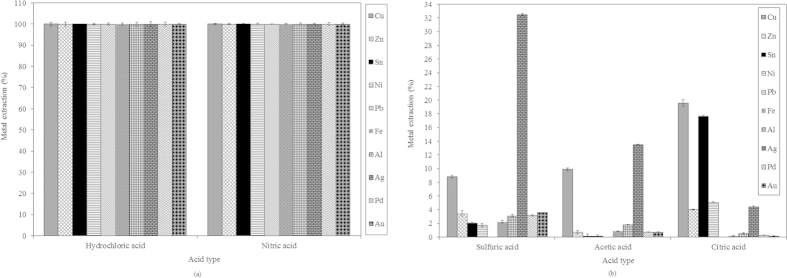
Amounts of metals recovered from a 4 cm × 4 cm PCB piece (a) by HCl and HNO_3_ and (b) by H_2_SO_4_, C_2_H_4_O_2_ and C_6_H_8_O_7_, at 150 rpm and room temperature.

**Figure 4:**
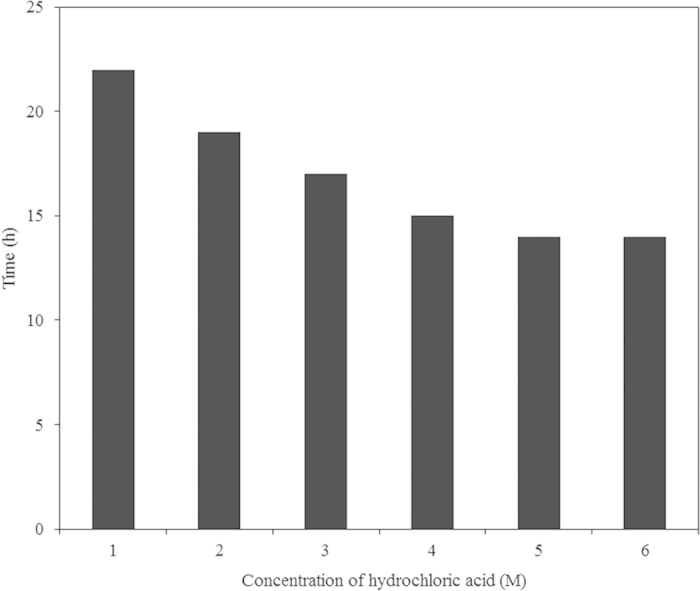
Effect of HCl concentration on the time required for complete metal recovery from a 4 cm × 4 cm PCB piece at 150 rpm and room temperature.

Significant work has been reported on the hydrometallurgical recovery of metals from PCBs^1^,^22^,^23^. HNO_3_, HCl, and H_2_SO_4_ solutions are often used as leaching agents in hydrometallurgical treatments. Hydrometallurgical treatments have more flexibility during the upscaling and control processes^23^,^24^,^25^. Therefore, in the present study, five different acids, i.e., HCl, HNO_3_, H_2_SO_4_, acetic acid (C_2_H_4_O_2_) and citric acid (C_6_H_8_O_7_) were used as lixiviants for metal recovery from PCB pieces. It was observed that HCl and HNO_3_ removed all of the metal present on the PCB pieces (Fig. 2b). HCl required only 22 h to remove all of the metal from the PCB piece, whereas HNO_3_ took 96 h (Figs 2a and 3a,b). Visual examinations of samples treated with H_2_SO_4_, C_2_H_4_O_2_ and C_6_H_8_O_7_ showed poor metal recovery even after incubation for longer periods (Fig. 2b). The use of HCl is advantageous compared with the technique of Adhapure *et al.*^21^, who demonstrated complete metal recovery from a large PCB piece using a bioleaching process in 10 days (240 h). They also found poor lead solubilization, which caused the PCB pieces to show traces of solder, even after bioleaching. In the present study, there was no problem with lead solubilization. It was found that HCl solubilized 100% of the copper (Cu), zinc (Zn), tin (Sn), nickel (Ni), lead (Pb), iron (Fe), aluminum (Al), silver (Ag), gold (Au) and palladium (Pd) in 22 h from PCB pieces of size 4 cm × 4 cm (Fig. 3a). HNO_3_ also solubilized 100% of the metals, but this took 96 h (Fig. 3a). The H_2_SO_4_ (96 h), C_2_H_4_O_2_ (96 h) and C_6_H_8_O_7_ (364 h) treatments showed poor metal solubilization compared with HCl and HNO_3_, with only 8.8, 9.89 and 19.57% recovery of Cu achieved using H_2_SO_4_ (96 h), C_2_H_4_O_2_ (96 h) and C_6_H_8_O_7_ (364 h), respectively (Fig. 3b). In addition to the metal solubilization efficiency and time taken, the use of HNO_3_, H_2_SO_4_, C_2_H_4_O_2_ or C_6_H_8_O_7_ for metal leaching has several disadvantages. The cost of HNO_3_ is higher than that of other acids. The evolution of hazardous nitric oxides is unavoidable. In addition, after leaching, the HNO_3_ must be completely removed from the solution prior to metal separation, which makes the process complex and increases the cost^26^. H_2_SO_4_ appears to be a suitable lixiviant because of its low cost, but it forms a water insoluble species, PbSO_4_, when it reacts with Pb from the PCB piece^27^. In addition, in the present study, poor metal solubilization was observed using H_2_SO_4_. C_2_H_4_O_2_ and C_6_H_8_O_7_ are attractive leaching reagents. Their use is environmentally benign because the leaching is carried out at moderately acidic conditions, and they are biodegradable. However, they are not suitable leaching agents for large PCB pieces because they exhibit poor metal solubilization (Figs 2a,b and 4). They also suffer from low boiling and decomposition temperatures, and the recovery of heavy metals from these leach liquors is not easy^27^,^28^. According to Habbache *et al.*^28^, chloride, nitrate and sulfate ions are considered aggressive elements for metal removal. The high stability of chloro-complexes enhances the rate of metal dissolution. Nitrate and sulfate ions have different chemical characteristics, acting with lower strength. Therefore, even if they act in a similar manner to chloride ions, the rate of metal dissolution is lower. In contrast, the lack of anions that are able to enhance the dissolution rate of the metals might be responsible for the poor metal dissolution rate using organic acids^28^. In the present study, the concentration of all of the acids used for metal recovery was 1 M. The rough costs for preparing 1 M solutions of HCl, HNO_3_, H_2_SO_4_, C_2_H_4_O_2_ and C_6_H_8_O_7_ were 0.263, 0.400, 0.356, 0.239 and 1.460 USD, respectively. For a bioleaching process, Adhapure *et al.*^21^ incubated a 12 cm × 6 cm PCB piece with a mixed microbial culture in 1 L 9 K medium containing 45 g ferrous sulfate, which costs 2.93 USD. This process also required 10 days of incubation, which further increased the cost of the process due to the need for a constant supply of electricity. Therefore, the bioleaching process requires a larger capital investment than does the process proposed in the present study. The use of HCl for the recovery of metals from large PCB pieces is also more economical than the use of HNO_3_, H_2_SO_4_ or C_6_H_8_O_7_. Samina *et al.*^29^ suggested an order of corrosion activity for the various acids of HNO_3_ > H_2_SO_4_ > HCl > C_2_H_4_O_2_. This order shows that HCl is less corrosive than HNO_3_ and H_2_SO_4_. For industrial applications, corrosion-resistant materials can be used in the construction of the leaching equipment to avoid the corrosion caused by HCl. HCl has the ability to dissolve valuable metals from various sulfidic, oxidic and metallic resources^30^. Hence, HCl can be recognized as an effective lixiviant for the recovery of metals from large PCB pieces because of its selectivity and favorable economics. Considering these benefits, HCl was used to optimize the other process parameters.

The effect of the HCl concentration on the time required for complete metal recovery from PCBs was studied. Increasing the HCl concentration from 1 to 6 M significantly improved the metal recovery, and the time required for complete metal recovery decreased (Fig. 4). Above a concentration of 3 M, the variation in the time required for metal recovery is small. Considering this factor and safety reasons, a 1 M concentration of HCl was used in the subsequent studies.

**Figure 5:**
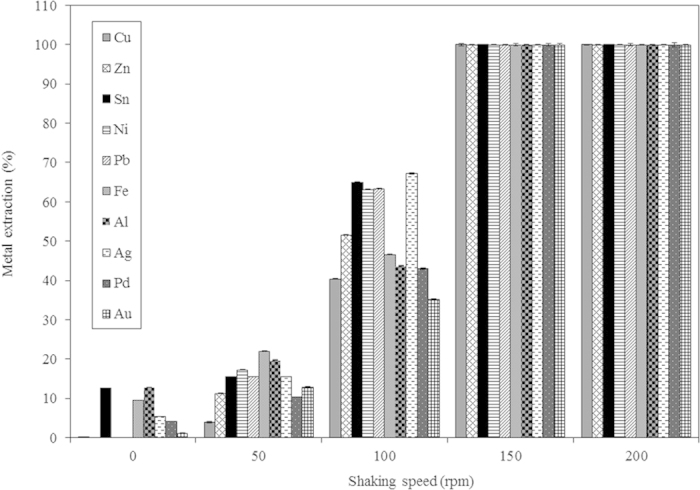
Effect of shaking speed on metal recovery from a 4 cm × 4 cm PCB piece after 22 h at room temperature.

The stirring speed showed a pronounced effect on metal recovery from PCB pieces. The metal recovery was poor at 0, 50 and 100 rpm. A 150 rpm stirring speed was found to be the optimum for complete metal recovery from PCB pieces (Fig. 5). According to Havlik *et al.*^24^, copper is significantly leached only in an oxidative environment. No significant reaction should occur between Cu and HCl because HCl is a non-oxidizing acid^24^. During the leaching process, the oxygen from the surrounding atmosphere enters the solution and acts as an oxidizing agent^24^:


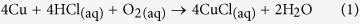


This might be a possible reason for the lower metal recovery from the PCB pieces at 0, 50 and 100 rpm. At 150 rpm, sufficient oxygen might have dissolved, thereby facilitating the complete metal recovery. A further increase in the stirring speed (200 rpm) showed similar results to those obtained at 150 rpm (Fig. 5). Therefore, a stirring speed of 150 rpm was maintained for the subsequent experiments to ensure the invariance of this parameter. Equations 2, 3, 4, 5, 6, 7, 8, 9, 10, 11 shows the possible reactions occurring for the other metals during the leaching process. Zn, Sn, Ni, Fe, Al and Ag metals react immediately with HCl to form metal chlorides^9^.


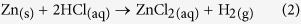



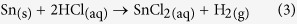



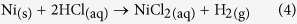



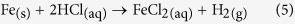



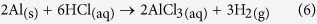



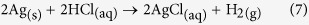


Xiu *et al.*^9^ suggested that Pb forms an insoluble species (PbCl_2_) when it reacts with HCl. However, Pb can still be leached by applying HCl. According to Zhang *et al.*^31^, Pb forms differently coordinated Pb^2+^ complexes with Cl, as per the following reaction:
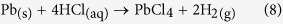


Au has little solubility in HCl. However, Au metal dissolves in the presence of an oxidant (air) and a halide (Cl_2_ in the present study) anion under highly acidic conditions^32^.


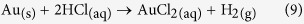


In HCl, Pd dissolves slowly in the presence of air. The oxygen from the air can react with the HCl through the following equation to form Cl_2_:


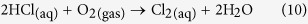


The formed Cl_2_ can then react with the Pd in the presence of HCl as per the following reaction:


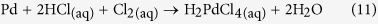


In this manner, Pd can form stable chloro-complexes in acidic chloride solutions. The formation of this complex dissolves the Pd^7^,^26^.

The effect of temperature on the metal leaching from PCBs is studied. The temperature has no effect on the metal leaching efficiency because similar metal leaching efficiencies were achieved for all of the temperatures used (room temperature-60 °C) (data not shown). These results contrast those obtained in previously published reports. Parhi *et al.*^30^ and Xiu *et al.*^9^ showed an increase in the metal leaching with an increase in temperature. The results of the present study are beneficial for a commercial process because they show that metal leaching is possible at low temperatures. Barik *et al.*^33^ suggested that higher temperatures lead to a substantial loss of valuable metals.

**Figure 6:**
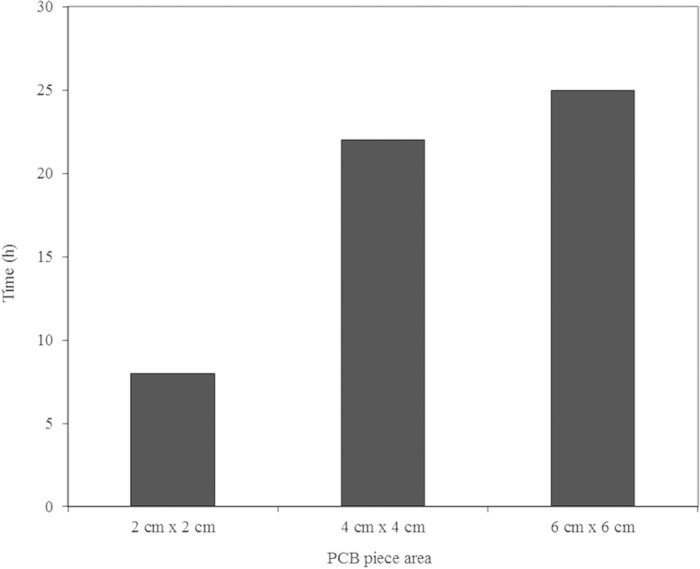
Effect of PCB piece size on time required for complete metal recovery at 150 rpm and room temperature.

Another experiment was carried out to study the effect of the size of the PCB piece on metal leaching performance. Here, 8 h was required for complete metal recovery from a 2 cm × 2 cm PCB piece. The time required for complete metal recovery increased with the size of the PCB piece; 22 and 25 h were required for 4 cm × 4 cm and 6 cm × 6 cm PCB pieces, respectively (Fig. 6). These results are more promising than those of Adhapure *et al.*^21^, who found that the metal recovery decreased with an increase in the size of the PCB piece. According to Adhapure *et al.*^21^, the use of PCB pieces instead of powder for metal recovery is quite simple and eliminates the cost of powder preparation, which further simplifies the recycling of the metal-free PCB pieces after the complete metal removal. Bioleaching processes that use PCB powder for metal recovery face precipitate formation problems. This precipitate is composed of Sn, Cu, Pb and Fe^21^,^34^. The formation of such a metal-containing precipitate contaminates the PCB powder, and it is difficult to separate the precipitate from the residual PCB powder^21^,^35^. When using microorganisms for the recovery of metals from PCBs, one should also consider the toxicity of the metal ions because metal toxicity can inhibit the growth of microorganisms and thereby the rate and degree of the oxidation of ferrous iron, which can further affect the metal dissolution process^36^. Electronic scrap has been reported to be alkaline in nature^37^. This phenomenon was not observed in distilled water but only occurred under conditions of low pH^36^. This might affect the bioleaching process at a high PCB powder dosage. Bioleaching processes based on ferrous ion oxidation may also face a jarosite precipitation problem, which would result in a decrease in the Fe^3+^ concentration in the solutions and cause low oxidation. It would also slow down the mass transfer by forming a layer on the surface of the PCB powder^36^. To avoid this, a low pH must be maintained. This requires the adaptation of microorganisms to a low pH environment^36^. The use of large pieces of PCBs overcomes all of these problems. From an industrial point of view, a higher treatment capacity can be achieved with lower investment using the method presented in this study. Further study is required for the purification and recovery of metals from pregnant leaching liquids. Various options are available for the purification and selective recovery of the metals from the pregnant leach liquor, including solvent extraction, adsorption on activated carbon, ion exchange, precipitation, cementation and electrowinning. The factors that affect the selection of an appropriate method are the leaching reagent system (e.g., Cl^−^, SO_4_^2−^), concentration of metal(s), impurities, and economy of the process. However, a very limited number of articles is available on the purification and recovery of individual metals from PCB leach liquor^1^,^38^.

## Conclusion

In the present study, hydrometallurgical leaching was applied to waste PCBs to design a metal recovery process. The results show that the hydrometallurgical recovery of metals from large pieces of waste PCBs is possible. The HCl took less time for the metal recovery and was thus a very effective leachant. The use of large pieces of PCBs for metal recovery will facilitate the recycling of the remaining boards. It will also avoid the problem of precipitate contamination when recovering the metals from the leach liquors. Hence, an economical hydrometallurgical process can be established for the recovery of metals from waste PCBs.

## Materials and Methods

All tests were performed in three replicates.

### Materials

Nitric acid (HNO_3_ 65%), sulfuric acid (H_2_SO_4_ 95%), hydrochloric acid (HCl 37%), citric acid (C_6_H_8_O_7_) and acetic acid (C_2_H_4_O_2_) were purchased from Sigma-Aldrich.

### Collection of PCBs

PCBs were collected from the local scrap market. Various components, including RAM, PCI slot, chip slots, integrated circuits (ICs), and gold-plated terminals of connectors, are attached to the substrate, depending on the specific functions and applications. These components also contain various precious metals^1^,^38^. These attached parts were removed manually from the PCBs to allow the removal of hazardous components and pre-concentration of precious metals^1^. These parts can be pulverized separately, and the obtained powder can be used for the recovery of precious metals using various methods. The PCBs were then cut into 4 × 4 cm pieces.

### Removal of chemical coating of PCBs

The PCBs have a chemical coating (solder mask), which is commonly made of epoxy. The solder mask covers the metals mounted on the PCBs and does not allow the leaching agent to penetrate through it for the recovery of metals. Adhapure *et al.*^21^ used 10 M sodium hydroxide (NaOH) to remove this chemical coating. This solution can facilitate the use of large pieces of PCBs for the recovery of metals and was applied in the present study. PCBs were dipped overnight in 10 M NaOH under static conditions. After the NaOH treatment, samples were taken and sent for ICP analysis to determine the concentrations of the dissolved metals in NaOH. The PCBs are then washed under running tap water, and the wash water was replaced by fresh distilled water until the adhered NaOH was removed, as monitored by a neutral pH reading of the wash water. The washed PCBs were then used for further study.

### Leaching procedure and conditions

Aqua regia was used to fully leach the metals from the PCB specimen. Following the filtration of the digestion solution, the metal concentrations were determined by inductively coupled plasma resonance spectroscopy (ICP), with the results shown in Table 1. A comparison of the leaching behavior of five lixiviants was made using HCl, HNO_3_, H_2_SO_4_, C_2_H_4_O_2_ and C_6_H_8_O_7_. For this study, a 1 M solution (100 ml) of each acid was used. Deionized-distilled water was used in the preparation of the acid solutions. The effect of the incubation time on the leaching performance of each acid was studied. The experiments were carried out by separately immersing the PCB pieces (4 × 4 cm) in 1 M leaching solutions (100 ml) of HCl, HNO_3_, H_2_SO_4_, C_2_H_4_O_2_ and C_6_H_8_O_7_ in 500 ml beakers. The beakers were incubated while covered on an orbital rotary shaker at 150 rpm and room temperature. The PCB pieces were removed after the respective leaching periods, and photographs of the PCB surfaces were taken. Small aliquots of the samples were also taken and sent for ICP analysis to determine the metal content. Further process optimization was carried out using HCl because the best results were obtained using HCl. The effect of the acid concentration on the time required for metal recovery from the PCB piece was studied. Leach solutions (100 ml) were prepared at the required concentration of HCl (1–6 M). The beakers were kept for incubation on an orbital rotary shaker at 150 rpm and room temperature. A study was carried out to find the effect of the rotation speed on the metal recovery. For this study, 100 ml of 1 M HCl solution was used. The PCB pieces were covered with HCl solution in 500 ml beakers, and these beakers were incubated in an orbital rotary shaker at 0–150 rpm at room temperature for 22 h. The effect of temperature on the metal recovery was studied. The PCB pieces were immersed in 100 ml of 1 M HCl solution in 500 ml beakers that were incubated at various temperatures (room temperature-60 °C) and 150 rpm for 22 h. The effect of the size of the PCB on the time required for metal recovery was also studied. PCB pieces with sizes of 2 cm × 2 cm, 4 cm × 4 cm and 6 cm × 6 cm were immersed in 100 ml of 1 M HCl solution in 500 ml beakers at 150 rpm and room temperature.

## Additional Information

**How to cite this article**: Jadhav, U. and Hocheng, H. Hydrometallurgical Recovery of Metals from Large Printed Circuit Board Pieces. *Sci. Rep.* **5**, 14574; doi: 10.1038/srep14574 (2015).

## Figures and Tables

**Table 1:** Metal content of a 4 cm × 4 cm PCB piece.

Sr. No.	Metal	Metal content (mg/g)
1	Cu	117.33 (±0.28)
2	Zn	28.97 (±0.81)
3	Sn	12.62 (±0.27)
4	Ni	10.41 (±0.45)
5	Pb	9.34 (±0.49)
6	Fe	0.62 (±0.006)
7	Al	0.325 (±0.004)
8	Ag	0.02 (±0.002)
9	Pd	0.012 (±0.0008)
10	Au	0.0075 (±0.0004)
